# The correlation between architecture and mRNA abundance in the genetic regulatory network of *Escherichia coli*

**DOI:** 10.1186/1752-0509-1-30

**Published:** 2007-07-17

**Authors:** Yohann Grondin, Derek J Raine, Vic Norris

**Affiliations:** 1 Centre for Interdisciplinary Science, Department of Physics and Astronomy, University of Leicester, Leicester LE1 7RH, UK; 2 Epigenomics Project, genopole^®^, 93 rue Henri-Rochefort, 91000 Evry, France; 3 Assemblages Moléculaires: Modélisation et Imagerie SIMS, FRE CNRS 2829, Faculté des Sciences et Techniques de Rouen, F76821 Mont Saint Aignan, France

## Abstract

**Background:**

Two aspects of genetic regulatory networks are the static architecture that describes the overall connectivity between the genes and the dynamics that describes the sequence of genes active at any one time as deduced from mRNA abundances. The nature of the relationship between these two aspects of these networks is a fundamental question. To address it, we have used the static architecture of the connectivity of the regulatory proteins of *Escherichia coli *to analyse their relationship to the abundance of the mRNAs encoding these proteins. In this we build on previous work which uses Boolean network models, but impose biological constraints that cannot be deduced from the mRNA abundances alone.

**Results:**

For a cell population of *E. coli*, we find that there is a strong and statistically significant linear dependence between the abundance of mRNA encoding a regulatory protein and the number of genes regulated by this protein. We use this result, together with the ratio of regulatory repressors to promoters, to simulate numerically a genetic regulatory network of a single cell. The resulting model exhibits similar correlations to that of *E. coli*.

**Conclusion:**

This analysis clarifies the relationship between the static architecture of a regulatory network and the consequences for the dynamics of its pattern of mRNA abundances. It also provides the constraints on the architecture required to construct a model network to simulate mRNA production.

## Background

The interactions of the many molecular constituents of a cell can be expressed in term of various networks, such as protein-protein interaction networks [1-3], metabolic networks [4,5] or genetic networks [6], in which the cell would be represented as a network of networks. Genetic regulatory networks are complex systems in which the agents or genes, that are the nodes of the network, each carry out the combined processes of transcription, translation and post-translational modifications, and the links represent the causal influences amongst these agents [7]. There are two aspects important for understanding these networks. The first is the static architecture. This comprises the overall connectivity or architecture, namely, which nodes are connected to which others, and the designation of links as either promoters or repressors. The second is the dynamics, namely, how it is determined which nodes are active at any one time, that is, the genes that are expressed, and what determines the level of activity at an active node. The architecture and dynamics gives rise to a pattern of activity over time and the corresponding time-dependent activities. In the case of cell biology, a major goal is to explain how the genetic regulatory network functions to produce mRNAs and hence phenotypes. Specific patterns of connection that are expected to reveal mechanisms of regulation and influence the dynamics of the network have already been shown [8,9]. However, there are several problems in trying to attain this goal on a larger scale. Indeed, the relationship between the static architecture and the dynamics of the genetic regulatory network is uncertain; in other words, the information available on the architecture, even if it were complete, may be insufficient to deduce the dynamics.

The information is unavailable that is needed on the distribution of the different species of mRNA over time in an individual cell and, moreover, that is needed for a representative number of the different cells that make up the heterogeneous population. On the other hand information is available on mRNA abundances in populations grown in a variety of conditions, but the relationship of these to the network architecture is unclear.

Network simulation might be expected to clarify the relationship between the phenotype of individual cells, the static architecture of their genetic regulatory circuits and the abundances of mRNA extracted from cell populations. Construction of such a model network should be constrained by the static architecture characteristic of real biological systems. For example, in both *Saccharomyces cerevisiae *and *Escherichia coli*, the number of regulatory proteins binding a gene is exponentially distributed, whilst the number of genes a transcription factor can bind follows a decaying power-law [6]. Another important constraint is the distribution of mRNA abundances in populations of cells which is best fitted by a log-normal function with a decaying power-law tail [10,11]. Here, we analyse the relationship between the network architecture and its regulatory behaviour using experimental data from *E. coli*. This reveals that the abundance of the mRNA encoding a regulatory protein is strongly correlated with the number of genes regulated by that protein.. To implement this relationship we use a model similar to a two state Boolean network in which nodes are either on or off, but with rates of production, for the nodes that are switched on, proportional to the number of outgoing links. Boolean networks have long been used as a biological model of cell differentiation [7,12] or in the inference of genetic regulatory networks from mRNA data [13-15], for example.

## Results

### Network architecture and mRNA abundances in *E. coli*

Information about the architecture of the transcriptional network of *E. coli *is contained in regulonDB [16] while data on mRNA abundances is extracted from the ASAP database [17]. Both sets of data are combined in order to investigate correlations between incoming or outgoing degrees of connectivity and mRNA abundances (see the methods section for details).

**Figure 1:**
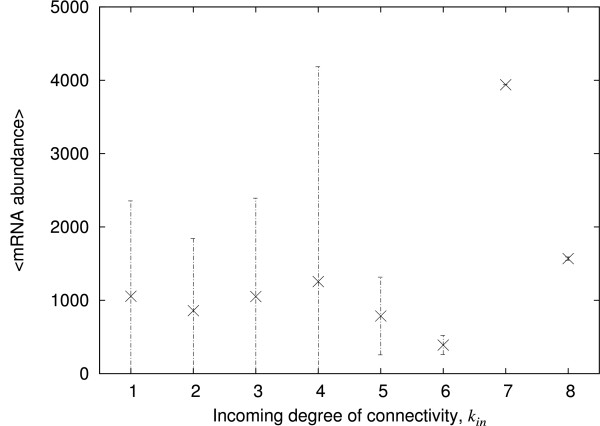
**The mRNA abundance versus incoming degrees of connectivity in *E. coli***. The relative mRNA abundances (on an arbitrary scale) as given by microarray experiments are averaged according to the incoming degree of the corresponding genes, that is the number of transcription factors regulating the given genes, and plotted against that degree. Only the genes that have an incoming degree greater than 0 have been selected, that is 787 genes. The error bars give the standard deviation.

**Figure 2:**
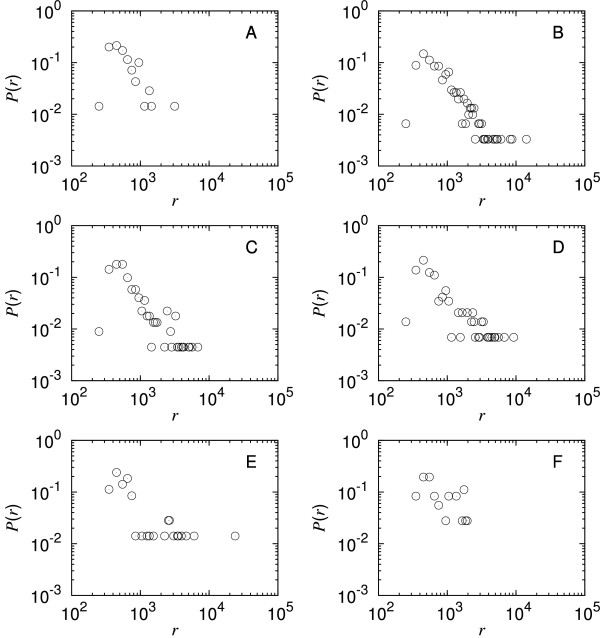
**Distribution of mRNA abundances per incoming degree of connectivity in *E. coli***. Each graph represents the distribution of mRNA abundance for a given incoming degree of connectivity, *k*_*in*_, that is the probability *P*(*r*) of finding a mRNA at abundance *r *for a given number of proteins regulating the corresponding genes. Graph A shows the distribution of mRNA abundance corresponding to the genes with *k*_*in *_= 1, graph B for *k*_*in *_= 2, graph C for *k*_*in *_= 3, graph D for *k*_*in *_= 4, graph E for *k*_*in *_= 5 and graph F for *k*_*in *_= 6. In graphs B, C and D the distribution of abundance appears clearly to follow a power-law tail which confirms the large standard deviation observed in Figure 1 when the mRNA abundance is averaged according to the incoming degree.

We look first at correlations between the incoming degree of connectivity of the genes and the level of abundance of the corresponding mRNA. The result presented in Figure 1 for the 787 selected genes (see the methods section) shows no evident linear correlation (Pearson correlation coefficient *r *= 0.01, p = ns; see additional file 1 for the corresponding scatter plot). The large standard deviations about most of the averaged abundances suggest that the abundance of mRNA, for a given incoming degree, is not normally distributed. This is confirmed in Figure 2 by plotting the distributions of mRNA abundances, for the incoming degrees of connectivity, *k*_*in*_, from 1 to 6. For intermediate degrees of connectivity, where the data are sufficient to reveal a trend, the distributions show decaying power-law tails, similarly to previous observations [10,11].

**Figure 3:**
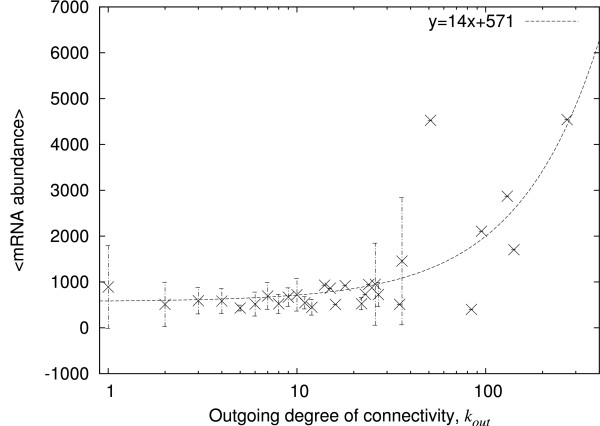
**Abundance of transcription factor versus their outgoing degree of connectivity in *E. coli***. The relative mRNA abundances (on an arbitrary scale) as given by microarray experiments are averaged according to the outgoing degree of the corresponding genes, that is the number genes the corresponding protein regulates, and plotted against that degree. Only the genes that have an outgoing degree greater than 0 have been selected, that is 113 genes. The error bars give the standard deviation.

We look then at whether the mRNA abundance of a transcription factor varies with the number of genes it regulates. The mRNA abundance of the corresponding transcription factors versus the outgoing degree of connectivity, shown in Figure 3, scatters over a larger range of connectivity than that of Figure 1 (see additional file 1 for the corresponding scatter plot). Most important is the linear correlation between the outgoing degree of connectivity and the average mRNA abundance (Pearson correlation coefficient *r *= 0.66, p < 0.001): the data indicate a trend for mRNA to be present at a relatively higher abundance when the corresponding transcription factor regulates a higher number of genes. Although suggestive, these data do not actually show that the patterns of incoming and outgoing connectivity in the static architecture are responsible for the similar patterns in the abundance of the mRNAs. This is because (i) the static architecture alone does not determine the dynamical behaviour of a network and (ii) the abundances are obtained from heterogeneous populations of cells. We have therefore turned to a model network in which the functioning of a network with concomitant production of mRNA is simulated at the level of individual 'bacteria'. However, we average the mRNA over a cycle, which is equivalent to averaging over a heterogeneous population.

### The network simulation

We have adopted a simple genetic regulatory model network in which the nodes are agents that carry out the combined processes of transcription, translation and post-translational modifications. The interactions between the agents are the cause of the actions performed by the agents. The indirect influence of one gene on another in a cell is therefore replaced by the direct action of one agent on another in the model. This simplification, which allows us to concentrate only on the mRNA abundance, is valid if we consider the mRNA abundance to be correlated to the protein abundance. Such correlation has been previously studied in *S. cerevisiae *[18,19]. The model and the simulation from which we extract the following data are described in more detail in the methods section.

**Figure 4:**
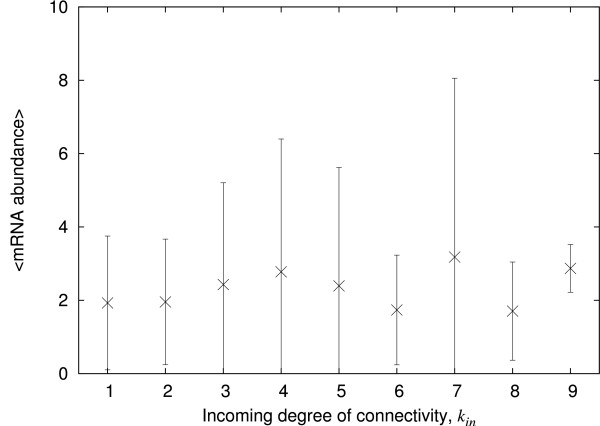
**Abundance of transcription factor versus their incoming degree of connectivity in the simulation**. The plotted mRNA abundance is the simulated mRNA abundance averaged over time for a given value of the incoming degree of connectivity of the corresponding nodes. Only the nodes that have been ON at least once during the recorded period and that have an incoming degree of connectivity greater than 0 have been recorded, in this case 732 nodes. The error bars give the standard deviation.

The mRNA abundances generated at the nodes of our model network are averaged over time and plotted against the incoming degree of connectivity of the corresponding nodes. Figure 4 shows that the result is similar to that observed in *E. coli*, with no evident correlation between the abundance and the degree of connectivity (Pearson correlation coefficient *r *= 0.06, p = ns; see additional file 1 for the corresponding scatter plot). A large standard deviation about the mean abundance is also observed. The distribution of abundances at each degree, where there are enough data to display a trend, exhibits a decaying power-law tail like the *E. coli *data in Figure 4 (data not shown).

**Figure 5:**
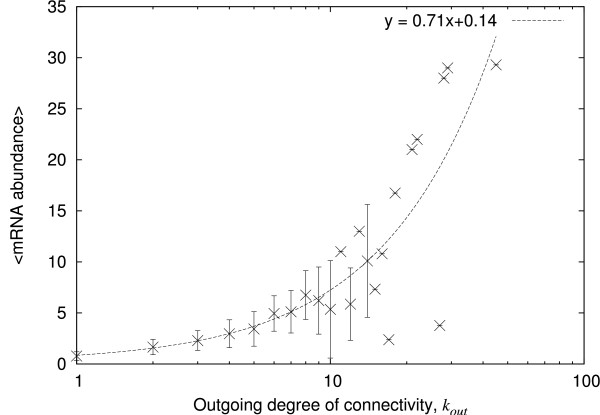
**Abundance of transcription factor versus their outgoing degree of connectivity in the simulation**. The plotted abundance is the simulated mRNA abundance averaged over time at a given value of the outgoing degree of connectivity of the corresponding node. Only the nodes that have been ON at least once during the recorded period and that have an outgoing degree of connectivity greater than 0 have been recorded, in this case 730 nodes. The error bars give the standard deviation.

The simulated mRNA abundances, averaged over time for nodes of a given outgoing degree of connectivity, show a linear dependency on the outgoing degree of the nodes (Pearson correlation coefficient gives *r *= 0.86, p < 0.001; Figure 5 and additional file 1 for the scatter plot). This result for outgoing degree of connectivity, like that for the incoming degrees of connectivity, is similar to that found in the *E. coli *data.

## Discussion

The approach to the cell as a network – or network of networks – holds out the promise of a deep level of understanding of the origin of the phenotype. Study of the connectivity in metabolism has revealed power-law relationships [4,5], the significance of which is a matter of debate [20,21], whilst study of the connectivity of the transcriptional regulatory network in *S. cerevisiae *has revealed that the number of genes encoding transcription factors has a power-law relationship to the degree of outgoing connections (how many genes are regulated by the transcription factor) but has an exponential relationship to the incoming connections (how many transcription factors regulate the gene in question) [6]. This raises the question of the relationship between such static patterns in the architecture of the overall network and the phenotype in terms of the mRNA of individual cells in which only a part of the network functions at any one time.

To begin to address it, we looked first at the static architecture of the network of regulatory proteins in *E. coli *from the RegulonDB [16] and at the abundances of the mRNA corresponding to these proteins in the ASAP database [17]. We find that there is a linear relationship between the outgoing degree of connectivity of the regulatory protein and the abundance of mRNA encoding that protein, but that there is no evident relationship between the incoming degree and mRNA abundance. It might be argued that this has little meaning. On the one hand, the ASAP data are of heterogeneous populations of cells and not of individuals (even if mainly one set of growth conditions was used) whilst, on the other hand, the similar pattern of outgoing degrees of connectivity observed in architecture by others and in mRNA abundances by us here might be coincidental. We therefore constructed an artificial network based closely on the architecture of the genetic regulatory network of *E. coli*. The running of this network and the accumulation of the 'mRNA' generated is equivalent to taking a series of snapshots of an individual bacterium and adding up all the mRNA generated (which *in vivo *generally has a short half-life). Comparison of the results from the simulation data with those from *E. coli *populations indicates similar behaviour in terms of correlations between the mRNA abundances and the architecture of the system. It might be argued that this result is to be expected since the production of mRNA built into the model is correlated to the outgoing degree of the node. However, this argument ignores the fact that it is the dynamics of the network that determines which nodes are actually activated. Indeed, it is this relationship between static architecture and functional dynamics that the model network clarifies.

In comparison with the *E. coli *data, the Pearson correlation coefficient for the simulated data reveals more dramatically the fact that mRNA abundance is correlated to the outgoing rather than the incoming degree of connectivity. This may be because, in the model network, the gene-to-gene interactions are represented by a single intermediate, as we have assumed the mRNA abundances to be perfectly correlated to that of the proteins. Assuming a weaker connection would simply tend to diminish the correlation with the degree of connectivity.

The proportion of negative links *μ *plays a role similar to that of the homogeneity parameter in Boolean network [7,22], in that it determines the probability for a node to be ON according to its inputs. It is easy to see that for *μ *close to zero, most of the nodes in the network are ON and the distribution of abundance must then be very similar to the distribution of the outgoing degrees of connectivity (equation (2)). In this case the correlation, between the outgoing degree of connectivity and the abundance, exhibited in the dynamical behaviour of the network, follows from the static architecture and is close to 1. On the other hand, for *μ *close to 1, as most of the nodes are OFF, the correlation is close to 0. In this case the static architecture is unrelated to the mRNA abundance! Furthermore, it is also possible to engineer the distribution of negative links so that, for example, the probability that the nodes of high outgoing degree of connectivity are expressed tends to zero. This would have a similar effect on the correlation as an increase in *μ*.

In our model, the proportion of negative links is therefore an essential parameter in determining the degree of the correlation. Thus it is a matter for numerical experiment to determine the correlation for a realistic range of values of *μ*. We find that for 0 < μ < 1, the correlation *r *is 1 <*r *< 0. At *μ *= 0.37 as shown in the illustration, the correlations are closed to what is observed in *E. coli*.

Many factors intervene in the dynamics of gene regulation. This includes local factors such as the sequence specificity of the transcription factor DNA binding site [23] and global ones such as the structural organisation of the chromosomes [24]. The timing of interaction is another important factor: differential timing of interaction is suggested to explain the large diversity of organisms against a not so large genomic diversity [25]. Furthermore, it is also known that local structures, such as the feed-forward loop significantly represented in *E. coli *for example, have an effect on the kinetics of interaction which can affect the regulatory response of genes [9,26]. The correlation we find between the architecture of the network and the mRNA activity is one of the numerous factors influencing gene regulation and needs to be considered as such.

We have shown that there is a significant correlation between architecture and mRNA. We can ask the reason for such correlation. We speculate that it may have to do with a selective pressure to produce sufficient regulator for the task of regulation. Producing too little leads to failure to generate the phenotype whilst producing too much is not simply wasteful but also means that there is more regulator to be eliminated in order to generate another phenotype.

The mRNA abundance is obviously correlated to the mechanism of regulation of transcription factors. Here we have shown that there is also a significant correlation between the architecture and the function.

One value of simulated genetic regulatory networks is that they can help bridge gaps in our understanding, such as that between the static architecture of the biological network and the consequences of its dynamic functioning in individual cells (which results in the experimentally accessible data on heterogeneous populations of mRNA). The model network used here is based closely on information about the architecture of the real network. Determining what information is needed to go from architecture to functioning and back will depend on continued exploration of real and simulated networks and of the relationship between them.

## Conclusion

Analysis of experimental data from *E. coli *suggests a significant correlation between the number of genes regulated by a transcription factor and the abundance of the mRNA that encode this transcription factor. It does not suggest an evident correlation between the number of regulators of a gene and the abundance of the mRNA it encodes. Since the relationship between the architecture of a genetic regulatory network and its functioning is unclear, a model network was constructed with architecture similar to that of the *E. coli *network. The correlations between mRNA abundances and degrees of incoming and outgoing connectivity observed in the *E. coli *data are corroborated by the correlations in the data generated by the model.

## Methods

### The *E. coli *data

The regulonDB [16] gives for *E. coli *the regulatory genes and the genes that they regulate. We calculate the incoming degree of connectivity, *k*_*in*_, of given genes corresponding to the number of transcription factors regulating a given gene, as well as the outgoing degree of connectivity, *k*_*out*_, which corresponds to the number of genes a given transcription factor regulates. In July 2005, this database contained 131 regulatory genes and 925 regulated genes.

The mRNA abundances in *E. coli *is obtained from the ASAP database [17]. The abundances were measured in microarray experiments using mRNAs extracted from populations of *E. coli *grown in standard conditions in which cells were grown for the majority in MOPS minimal medium and harvested in early exponential phase [27]. Here, the mRNA abundances we use are the average values over the 8 to 11 repeats of the microarray experiments.

By combining the two datasets we identify 859 genes and for which the abundance and the degrees of incoming and outgoing connectivity are known (see additional files 2 and 3 for the data). Amongst those genes, of which 113 are regulatory, 72 are not associated to regulatory genes (*k*_*in *_= 0) and 787 are regulated (*k*_*in *_> 0). The genes are then grouped according their incoming or outgoing degree of connectivity and the average of the corresponding mRNAs calculated.

### Characteristics of the model

In our genetic regulatory model, we consider directed networks where the agents, or nodes, represent the cellular machinery and the links represent the regulating influence of the agents on each other. This model is based on a Boolean network [7,12,15]. The three principal features of this model described below are (i) the architecture of the network, (ii) the dynamics of regulation of the agents and (iii) the activity function of the agents.

The network is represented by its adjacency matrix *A*, with elements *a*_*ij *_given by

aij={0, if there  is no link from the node j to the node  i;                                    1, if node j is connected  and directed to node i and acts  as an inducer on i;-1, if node j is connected  and directed to node i and acts as a repressor  oni.
 MathType@MTEF@5@5@+=feaafiart1ev1aaatCvAUfKttLearuWrP9MDH5MBPbIqV92AaeXatLxBI9gBaebbnrfifHhDYfgasaacH8akY=wiFfYdH8Gipec8Eeeu0xXdbba9frFj0=OqFfea0dXdd9vqai=hGuQ8kuc9pgc9s8qqaq=dirpe0xb9q8qiLsFr0=vr0=vr0dc8meaabaqaciaacaGaaeqabaqabeGadaaakeaacqWGHbqydaWgaaWcbaGaemyAaKMaemOAaOgabeaakiabg2da9maaceaabaqbaeqabmqaaaqaaiabbcdaWiabbYcaSiabbccaGiabbMgaPjabbAgaMjabbccaGiabbsha0jabbIgaOjabbwgaLjabbkhaYjabbwgaLjabbccaGiabbccaGiabbMgaPjabbohaZjabbccaGiabb6gaUjabb+gaVjabbccaGiabbYgaSjabbMgaPjabb6gaUjabbUgaRjabbccaGiabbAgaMjabbkhaYjabb+gaVjabb2gaTjabbccaGiabbsha0jabbIgaOjabbwgaLjabbccaGiabb6gaUjabb+gaVjabbsgaKjabbwgaLjabbccaGiabdQgaQjabbccaGiabbsha0jabb+gaVjabbccaGiabbsha0jabbIgaOjabbwgaLjabbccaGiabb6gaUjabb+gaVjabbsgaKjabbwgaLjabbccaGiabbccaGiabdMgaPjabbUda7iabbccaGiabbccaGiabbccaGiabbccaGiabbccaGiabbccaGiabbccaGiabbccaGiabbccaGiabbccaGiabbccaGiabbccaGiabbccaGiabbccaGiabbccaGiabbccaGiabbccaGiabbccaGiabbccaGiabbccaGiabbccaGiabbccaGiabbccaGiabbccaGiabbccaGiabbccaGiabbccaGiabbccaGiabbccaGiabbccaGiabbccaGiabbccaGiabbccaGiabbccaGiabbccaGiabbccaGaqaaiabbgdaXiabbYcaSiabbccaGiabbMgaPjabbAgaMjabbccaGiabb6gaUjabb+gaVjabbsgaKjabbwgaLjabbccaGiabdQgaQjabbccaGiabbMgaPjabbohaZjabbccaGiabbogaJjabb+gaVjabb6gaUjabb6gaUjabbwgaLjabbogaJjabbsha0jabbwgaLjabbsgaKjabbccaGiabbccaGiabbggaHjabb6gaUjabbsgaKjabbccaGiabbsgaKjabbMgaPjabbkhaYjabbwgaLjabbogaJjabbsha0jabbwgaLjabbsgaKjabbccaGiabbsha0jabb+gaVjabbccaGiabb6gaUjabb+gaVjabbsgaKjabbwgaLjabbccaGiabdMgaPjabbccaGiabbggaHjabb6gaUjabbsgaKjabbccaGiabbggaHjabbogaJjabbsha0jabbohaZjabbccaGiabbccaGiabbggaHjabbohaZjabbccaGiabbggaHjabb6gaUjabbccaGiabbMgaPjabb6gaUjabbsgaKjabbwha1jabbogaJjabbwgaLjabbkhaYjabbccaGiabb+gaVjabb6gaUjabbccaGiabdMgaPjabbUda7aqaaiabd2caTiabbgdaXiabbYcaSiabbccaGiabbMgaPjabbAgaMjabbccaGiabb6gaUjabb+gaVjabbsgaKjabbwgaLjabbccaGiabdQgaQjabbccaGiabbMgaPjabbohaZjabbccaGiabbogaJjabb+gaVjabb6gaUjabb6gaUjabbwgaLjabbogaJjabbsha0jabbwgaLjabbsgaKjabbccaGiabbccaGiabbggaHjabb6gaUjabbsgaKjabbccaGiabbsgaKjabbMgaPjabbkhaYjabbwgaLjabbogaJjabbsha0jabbwgaLjabbsgaKjabbccaGiabbsha0jabb+gaVjabbccaGiabb6gaUjabb+gaVjabbsgaKjabbwgaLjabbccaGiabdMgaPjabbccaGiabbggaHjabb6gaUjabbsgaKjabbccaGiabbggaHjabbogaJjabbsha0jabbohaZjabbccaGiabbggaHjabbohaZjabbccaGiabbggaHjabbccaGiabbkhaYjabbwgaLjabbchaWjabbkhaYjabbwgaLjabbohaZjabbohaZjabb+gaVjabbkhaYjabbccaGiabbccaGiabb+gaVjabb6gaUjabdccaGiabdMgaPjabd6caUaaaaiaawUhaaaaa@4FDB@

Contrary to the classical representation of Boolean networks, the links between agents are considered to be either positive or negative. This models, respectively, the activation or inhibition capability of the agents on each other. The proportion of negative links is labelled *μ*. The difference from standard Boolean networks is in the proportion of negative links *μ *which differs from what is called the internal homogeneity, *p*, that is the proportion of output nodes that are ON according the inputs [7,22].

Data show that in *S. cerevisiae *and *E. coli *[6] the distribution of the incoming degree of connectivity follows a Poisson distribution while the distribution of the outgoing degree of connectivity follows a power-law. To generate a network with such architecture, we first generate the adjacency matrix of an undirected network, that is *a*_*ij *_= *a*_*ji*_, with a power-law distribution of both the incoming and outgoing degree of connectivity using the Barabasi-Albert model [28]. A direction is then given to the links by setting at random element *a*_*ij *_= 0 or *a*_*ji *_= 0 with equal probability. The columns of the resulting adjacency matrix are then randomised in order to give a Poisson distribution to the incoming degree of connectivity while the distribution of the outgoing degree of connectivity remains unchanged.

As for classical Boolean networks, each agent is characterised by its binary state. The configuration of the network at any one time is given by the vector *S*(*t*) where the element *s*_*i*_(*t*) is the state of agent *i *at time *t*, such that *s*_*i*_(*t*) = 0 if the agent is OFF; otherwise *s*_*i*_(*t*) = 1 and the agent is ON. The dynamics of the network is provided by a simple rule in which the state of the agents at a given time depends only on the configuration of the network at the previous time. This rule states that a node is ON if the number of activated positive incoming links is greater than the number of negative ones plus a bias of activation, *b*_*a*_. Thus only the nodes that are ON can exert their control over the other nodes. This translates to the following expression in which a node *i *is ON if

∑jaijsj(t)+ba>0
 MathType@MTEF@5@5@+=feaafiart1ev1aaatCvAUfKttLearuWrP9MDH5MBPbIqV92AaeXatLxBI9gBaebbnrfifHhDYfgasaacH8akY=wiFfYdH8Gipec8Eeeu0xXdbba9frFj0=OqFfea0dXdd9vqai=hGuQ8kuc9pgc9s8qqaq=dirpe0xb9q8qiLsFr0=vr0=vr0dc8meaabaqaciaacaGaaeqabaqabeGadaaakeaadaaeqbqaaiabdggaHnaaBaaaleaacqWGPbqAcqWGQbGAaeqaaOGaem4Cam3aaSbaaSqaaiabdQgaQbqabaGccqGGOaakcqWG0baDcqGGPaqkcqGHRaWkcqWGIbGydaWgaaWcbaGaemyyaegabeaakiabg6da+iabicdaWaWcbaGaemOAaOgabeqdcqGHris5aaaa@4030@

and it is OFF otherwise. In the following, *b*_*a *_is in fact set to 0. The activation function in equation (1) expresses thresholds conditioned, for example, by the specificity of the sequence or the concentration of the regulator, which may be more realistic than the binary binding or not-binding of a transcription factor to specific DNA sequences [23,29]. Thus, although equation (1) could be expressed in terms of rather complex Boolean functions the direct formulation given here is more appropriate.

We add to the model that the level of expression of an agent is measured by the abundance of its product and is given by the activity function. We do not consider here the effects of a variable lifetime of the product, which is then arbitrarily set to one time step. Under this condition, the rate of synthesis of a product and its abundance are identical. The abundance is proportional to the rate of transcription in two cases: (i) the reaction is at equilibrium or, (ii) as here, the product is quickly degraded (1 time step in our case).

The study of correlations between abundance and degree of connectivity in *E. coli *suggests a linear correlation between the outgoing degree of connectivity and the mRNA abundance. The product abundance related to the activity of an agent *i *at *t*, X_*i*_(*t*), is therefore expressed as

Xi(t)=si(t)∑j|aji|+be,
 MathType@MTEF@5@5@+=feaafiart1ev1aaatCvAUfKttLearuWrP9MDH5MBPbIqV92AaeXatLxBI9gBaebbnrfifHhDYfgasaacH8akY=wiFfYdH8Gipec8Eeeu0xXdbba9frFj0=OqFfea0dXdd9vqai=hGuQ8kuc9pgc9s8qqaq=dirpe0xb9q8qiLsFr0=vr0=vr0dc8meaabaqaciaacaGaaeqabaqabeGadaaakeaacqWGybawdaWgaaWcbaGaemyAaKgabeaakmaabmaabaGaemiDaqhacaGLOaGaayzkaaGaeyypa0Jaem4Cam3aaSbaaSqaaiabdMgaPbqabaGcdaqadaqaaiabdsha0bGaayjkaiaawMcaamaaqafabaWaaqWaaeaacqWGHbqydaWgaaWcbaGaemOAaOMaemyAaKgabeaaaOGaay5bSlaawIa7aiabgUcaRiabdkgaInaaBaaaleaacqWGLbqzaeqaaaqaaiabdQgaQbqab0GaeyyeIuoakiabcYcaSaaa@48D8@

where *b*_*e *_is the bias of the level of expression or base level at which an agent is expressed when

si(t)∑j|aji|=0.
 MathType@MTEF@5@5@+=feaafiart1ev1aaatCvAUfKttLearuWrP9MDH5MBPbIqV92AaeXatLxBI9gBaebbnrfifHhDYfgasaacH8akY=wiFfYdH8Gipec8Eeeu0xXdbba9frFj0=OqFfea0dXdd9vqai=hGuQ8kuc9pgc9s8qqaq=dirpe0xb9q8qiLsFr0=vr0=vr0dc8meaabaqaciaacaGaaeqabaqabeGadaaakeaacqWGZbWCdaWgaaWcbaGaemyAaKgabeaakiabcIcaOiabdsha0jabcMcaPmaaqafabaWaaqWaaeaacqWGHbqydaWgaaWcbaGaemOAaOMaemyAaKgabeaaaOGaay5bSlaawIa7aiabg2da9iabicdaWaWcbaGaemOAaOgabeqdcqGHris5aOGaeiOla4caaa@408C@

In the following, *b*_*e *_is set to 0.

The results of a typical run of the model are presented in the results section for a network with the following parameters. The network is constructed with 1500 nodes. This is larger than the present dataset used for *E. coli *but smaller than the 3000 estimated genes in *E. coli*. The proportion of negative links is set to *μ *= 0.37, close to the proportion of links that have a negative effect in *E. coli *(~0.41) as calculated from regulonDB. Note that the possibility of a link having dual actions (positive and negative), as observed in the data from *E. coli*, is not considered, hence there would be no significance to taking *μ *to be exactly 0.41. The results for *μ *= 0.37 are typical of the range 0.35 < μ < 0.42.

Genetic regulatory networks are sparse and the number of regulators acting on a gene is low [6]. Here, the mean degree of connectivity is set to 6 that is, on average, a node has 3 incoming and 3 outgoing links. For comparison, the mean degree of connectivity of the 113 regulatory genes in *E. coli *is about 15 while that of all the 859 genes is about 2. Finally, the networks are not autonomous and a number of nodes are therefore chosen to receive an external input. Those nodes remain ON at any time regardless the value of equation (1). In the present case, 50 nodes are chosen at random to receive an external input. This value is sufficient to ensure a dynamical response of the network without a strong clamping effect.

Because the dynamics of the model is that of Boolean networks, there is flexibility in the setting of the parameters without affecting the outcome. This ensures that the results are consistent over small variations of the parameters, upon further observations or collection of data, for example. This is also satisfactory with the incompleteness of the data, upon the condition that there are enough data to proceed at such a large scale. Therefore, the parameters used in the model need to be close to, but not necessarily equal to the set of observed parameters.

For the network architecture and with the parameters given above, simulations show that there is a large probability for the network to be periodic, an identical configuration of the network as given by the states of the nodes being likely to appear twice in a short length of time. Our model exhibits a period and has about a quarter of the nodes are ON permanently and another quarter where the state is variably ON and OFF. Population abundance data are generated from the network by summing over a period. In the simulation, we consider only the abundance at the nodes that have been activated at least once (see additional files 4 and 5 for the generated data and corresponding degrees of connectivity).

## Authors' contributions

The computing was carried out solely by YG. Otherwise the contributions were equal. All authors have read and approved the final manuscript.

## Supplementary Material

Additional file 1 Scatter plot of the data plotted in Figures 1, 2, 4 and 5. Each graph gives the scatter plot of the data presented in Figures 1, 2, 4 and 5. The Supplementary Figure 1 gives the scatter plot of the data plotted in Figure 1, the Supplementary Figure 2 the scatter plot of the data in Figure 3, the Supplementary Figure 3 the scatter plot of the data in Figure 4 and the Supplementary Figure 4 the scatter plot of the data in Figure 5. Click here for file

Additional file 2 Primary *E. coli *data for the correlation between the incoming degree and mRNA abundance. The first column corresponds to the incoming degree of connectivity of the genes in *E. coli *as given in RegulonDB (see the methods section for details). The second column gives the relative mRNA abundance of the corresponding genes obtained from the ASAP database (see the methods section for details). The mRNA abundances are the average of the relative value obtained from microarray experiments. Click here for file

Additional file 3 Primary *E. coli *data for the correlation between the outgoing degree and mRNA abundance. The first column corresponds to the outgoing degree of connectivity of the genes in *E. coli *as given in RegulonDB (see the methods section for details). The second column gives the relative mRNA abundance of the corresponding genes obtained from the ASAP database (see the methods section for details). The mRNA abundances are the average of the relative value obtained from microarray experiments. Click here for file

Additional file 4 Primary simulated data for the correlation between the incoming degree and mRNA abundance. The first column corresponds to the incoming degree of connectivity of the nodes in the model that have been at least ON once in the length of the period (see the methods section for details). The second column gives the 'mRNA' abundance averaged over the period of the corresponding nodes (see the methods section for details). Click here for file

Additional file 5 Primary simulated data for the correlation between the outgoing degree and mRNA abundance. The first column corresponds to the outgoing degree of connectivity of nodes in the model that have been at least ON once in the length of the period (see the methods section for details). The second column gives the 'mRNA' abundance averaged over the period of the corresponding nodes (see the methods section for details). Click here for file
