# Supplementary material for: The correlation between architecture and mRNA abundance in the genetic regulatory network of Escherichia coli
Source: BMC Syst Biol. 2007 Jul 17;1:30. doi: 10.1186/1752-0509-1-30 (PMC1940267; doi:10.1186/1752-0509-1-30)

Supplementary Figure 1 - Scatter plot of mRNA abundance versus incoming degrees of connectivity in *E. coli*.

The mRNA abundance as given by microarray experiments is plotted versus the incoming degree of the corresponding gene, that is the number of transcription factors regulating that gene. Only the genes that have an incoming degree greater than 0 have been selected, that is 787 genes. The mRNA abundances are given as the averaged value over the repeated microarray experiments (see the methods section for details).

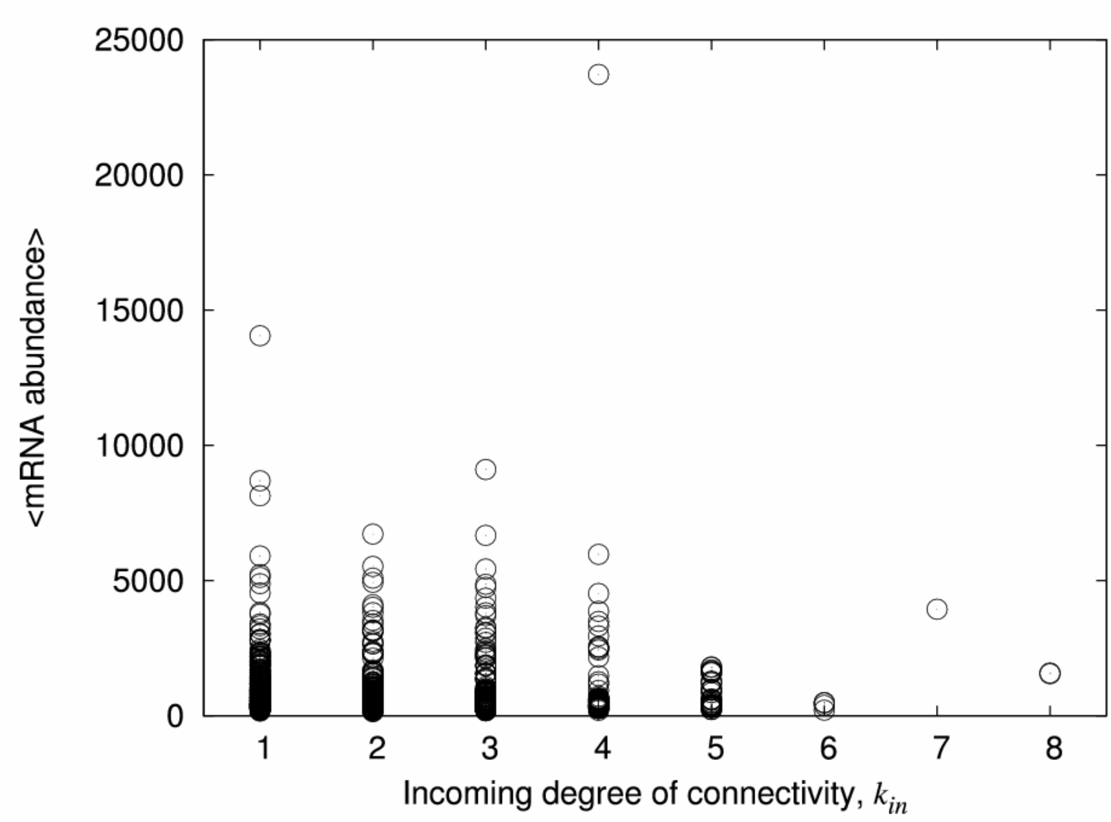

Supplementary Figure 2 - Scatter plot of mRNA abundance versus outgoing degrees of connectivity in *E. coli*.

The mRNA abundance as given by microarray experiments is plotted versus the outgoing degree of the corresponding gene, that is the number genes the corresponding protein regulates. Only the genes that have an outgoing degree greater than 0 have been selected, that is 113 genes. The mRNA abundances are given as the averaged value over the repeated microarray experiments (see the methods section for details).

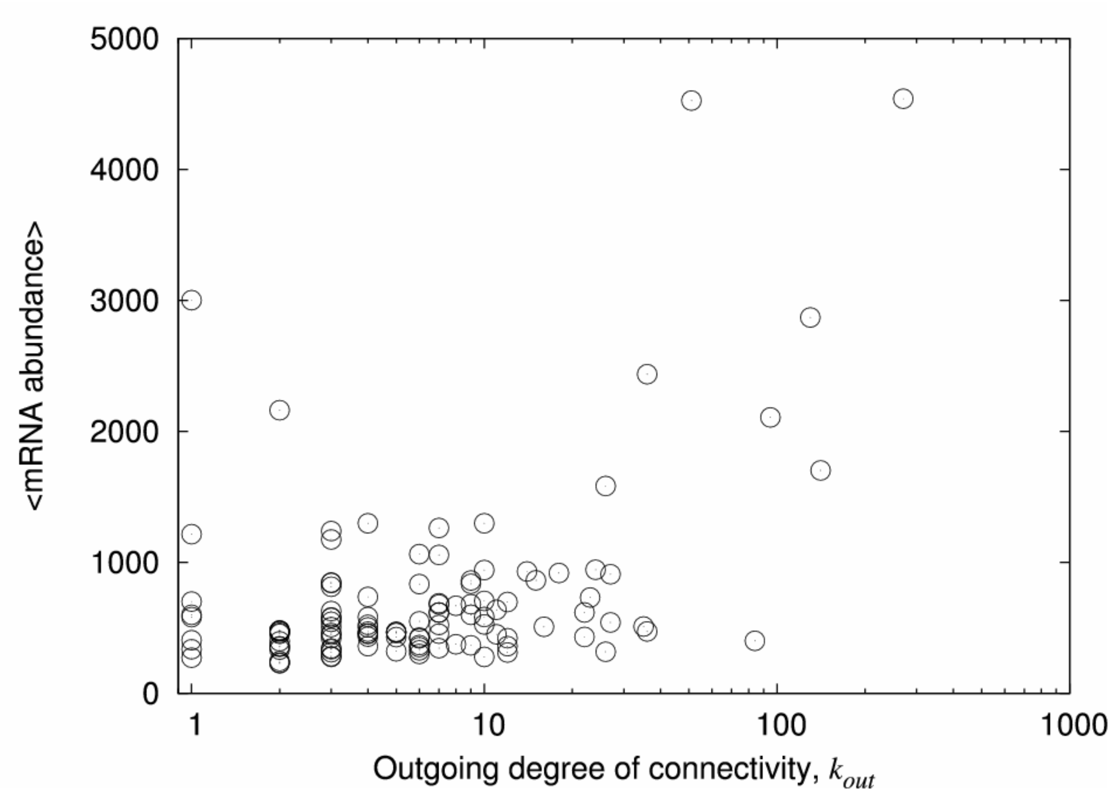

Supplementary Figure 3 - Scatter plot of the abundance of transcription factor versus their incoming degree of connectivity in the simulation.

Scatter plot of the simulated mRNA abundance averaged over time versus the incoming degree of connectivity of the corresponding nodes. Only the nodes that have been ON at least once during the recorded period and that have an incoming degree of connectivity greater than 0 have been recorded, in this case 732 nodes.

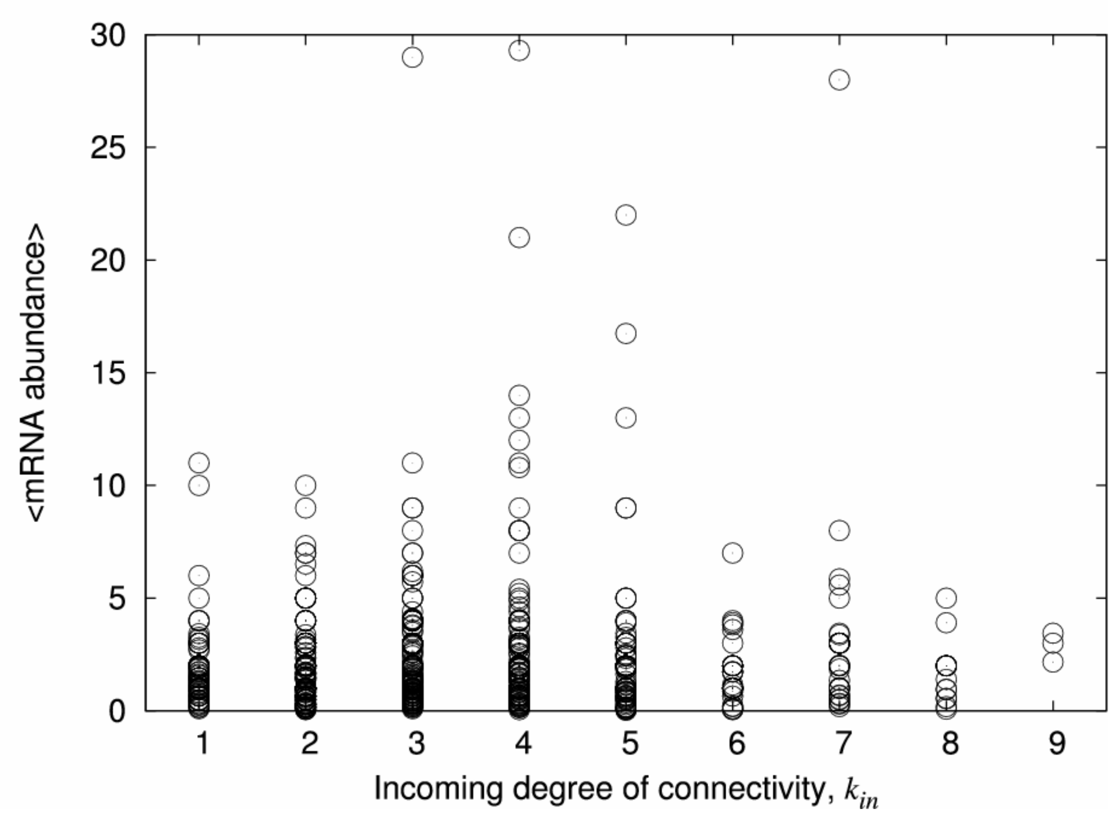

Supplementary Figure 4 - Scatter plot of the abundance of transcription factor versus their outgoing degree of connectivity in the simulation.

Scatter plot of the simulated mRNA abundance averaged over time versus the outgoing degree of connectivity of the corresponding nodes. Only the nodes that have been ON at least once during the recorded period and that have an outgoing degree of connectivity greater than 0 have been recorded, in this case 730 nodes.

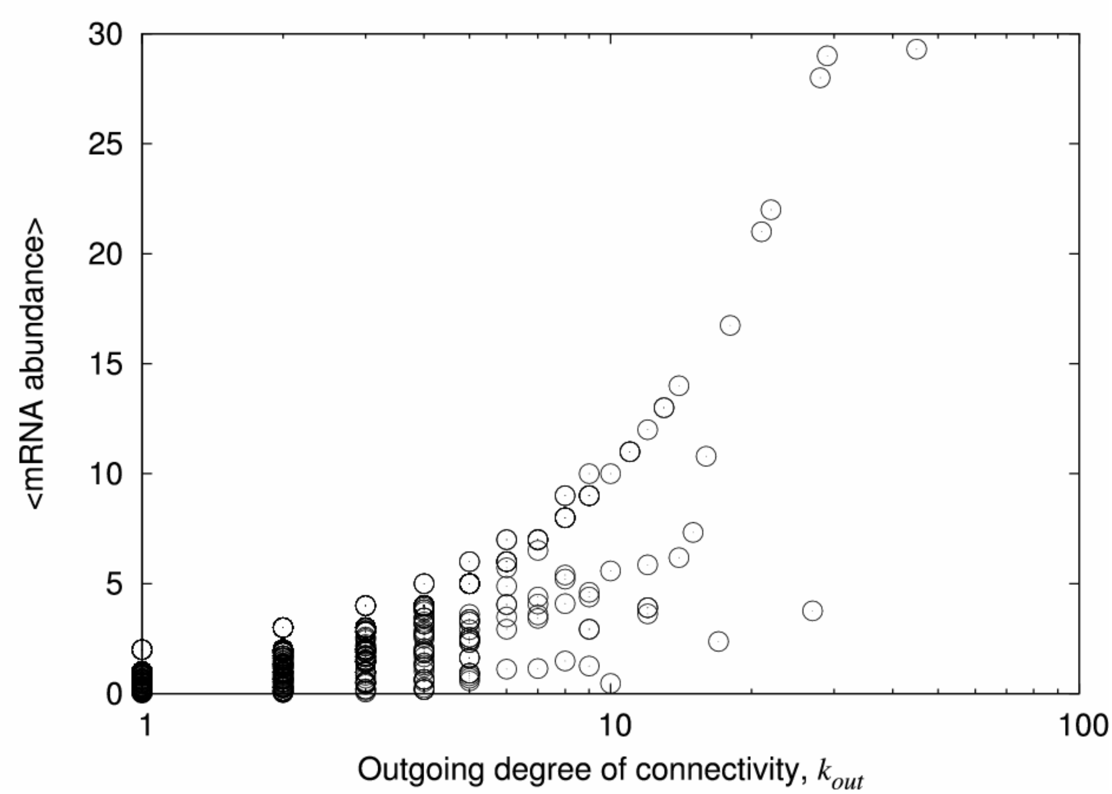

Supplement: Additional file 1 — Scatter plot of the data plotted in Figures 1, 2, 4 and 5. Each graph gives the scatter plot of the data presented in Figures 1, 2, 4 and 5. The Supplementary Figure 1 gives the scatter plot of the data plotted in Figure 1, the Supplementary Figure 2 the scatter plot of the data in Figure 3, the Supplementary Figure 3 the scatter plot of the data in Figure 4 and the Supplementary Figure 4 the scatter plot of the data in Figure 5. [file 1752-0509-1-30-S1.pdf]
